# Paget's bone disease in a nonendemic region: Exploring novel therapeutic strategies

**DOI:** 10.1002/ccr3.9364

**Published:** 2024-08-27

**Authors:** Nasrin Razavianzadeh, Soheil Shahramirad, Mohammad Hasani, Hessamedin Babaei

**Affiliations:** ^1^ Department of Medical Sciences, Shahrood Branch Islamic Azad University Shahrood Iran; ^2^ Student Research Committee, Shahrood Branch Islamic Azad University Shahrood Iran

**Keywords:** alendronate, Iran, Paget bone disease, treatment

## Abstract

Paget's disease of bone (PDB) is a chronic condition causing abnormal bone remodeling, leading to pain, fractures, and complications. A 57‐year‐old female patient, asymptomatic and devoid of pain, incidentally exhibited elevated levels of alkaline phosphatase. Following a thorough consideration of potential differential diagnoses, the eventual diagnosis established was PDB. We recommended Fosamax (70 mg alendronate tablets) at two tablets twice weekly for 3 months to manage PDB due to patient preference and side effects with intravenous zoledronic acid. Subsequent assessments of alkaline phosphatase levels during follow‐up examinations post‐treatment revealed a reduction in their values.

## 1 INTRODUCTION

Paget's disease is a prevalent bone disorder that ranks second in terms of frequency, following osteoporosis. Paget's disease is characterized by a nonmalignant and focal nature. The exact etiology of this condition remains unknown. However, it is widely acknowledged that Paget's disease is associated with irregularities and imbalances in bone production and turnover.^1^, ^2^ The pathophysiology of Paget's disease involves dysregulation in bone remodeling processes, primarily characterized by heightened bone resorption mediated by osteoclasts and excessive bone regeneration facilitated by osteoblasts.^3^ The prevalence of Paget's disease within the population of Northwest Europe, particularly in Britain, is estimated to be approximately 3%–5% among individuals aged 55 years and older. Moreover, the prevalence of Paget's disease remains elevated among immigrants who have migrated from Northwest European countries to regions such as Australia, New Zealand, and the United States.^4^ The incidence of Paget's disease tends to be higher in males compared to females. Additionally, the risk of developing Paget's disease increases with age.^2^ The etiology of Paget's disease involves a multifactorial interplay between genetic and environmental factors, including viral infections. Genetic predisposition has been identified as a significant contributor to the occurrence of the disease. Specific genetic mutations, such as SQSTM1 and TNFRSF11A, have been associated with an increased susceptibility to Paget's disease.^4^ It has been observed that approximately 15% of individuals diagnosed with Paget's disease exhibit a familial predisposition.^5^ The deformities resulting from Paget's disease have the potential to induce pathological fractures within the affected bones, as well as secondary osteoarthritis. It is noteworthy that Paget's disease often presents as either asymptomatic or with the manifestation of bone pain as the primary symptom.^6^ It exhibits a predilection for both solitary and multiple bone involvement, with the pelvis, vertebrae, skull, femur, and tibia being commonly affected sites. Notably, the disease is characterized by distinctive skeletal deformities, aberrant bone morphology, and augmented bone dimensions.^7^, ^8^ In certain instances, the tibia and femur bones may display bowing, resulting in heightened tolerance to bone and muscle pain. Moreover, engagement of the vertebral column can precipitate expansion and compression fractures, thereby eliciting back pain and kyphosis. Additionally, cranial alterations can contribute to the onset of headaches and hearing impairment.^8^, ^9^ Radiographic findings and specific views are essential for diagnosing and monitoring osteolysis in long bones. A distinct radiographic feature is a wedge‐shaped area of radiolucency extending from the diaphysis to the metaphysis, resembling a flame or blade of grass.^10^, ^11^ As the disease progresses, sclerotic lesions may exhibit a combination of lytic and sclerotic components, along with expansion, cortical thickening, and trabecular changes. Osteosclerosis primarily affects the inner table, resulting in thickening and irregular areas of sclerosis within the diploe. This leads to the formation of discrete islands of osteosclerosis, giving rise to the characteristic “cotton wool” appearance.^2^, ^12^ These radiographic findings provide valuable information for diagnosis, monitoring, and assessing treatment effectiveness.

## 2 CASE HISTORY/EXAMINATION

A 57‐year‐old female was referred to an internal medicine office for an incidental finding of extreme levels of alkaline phosphatase (ALP) in the laboratory evaluation due to her insistence for a history of 2 kg unintentional weight loss during the past 5 months.

She reported no jaundice, pruritus, B symptoms, alteration of bowel habits, nipple discharge, or vaginal bleeding. A thorough pertinent systemic review failed to uncover any history of recent or an insufficiency bone fracture, bone and joint pain, visual disturbance, hearing impairment, headache, numbness, or tingling.

Her medical history was remarkable for prediabetes mellitus and dyslipidemia, both of which were well controlled with Metformin 500 mg daily and Atorvastatin 10 mg daily.

Four years ago, she contracted the SARS‐CoV‐2 virus and administered 3 doses of Sinopharm BIBP COVID‐19 vaccine. About 5 years ago, she underwent a cholecystectomy. At age 51, she entered menopause. She came down with measles along with her older brother and younger sister during her childhood; one of her sisters who is 3 years younger had multiple insufficiency fractures and experienced the last one about 4 years prior to the current presentation which were never evaluated properly, and another sister was diagnosed and died of breast cancer at about the age of 28, therefore she was routinely screened for breast cancer. She was born and raised in a Persian family with no history of gout or bone cancer, never smoked, never got exposed to pesticides and radiation, and the patient doesn't report alcohol consumption.

On physical examination, the patient was a curious middle‐aged overweight well‐dressed lady with no evident skeletal deformity who walked with a normal gait without any trouble to the office. She was afebrile, pulse rate was 80 beats per minute, blood pressure 130/80 mmHg, and respiratory rate 18 breaths per minute and was unlaboured. The cervical and supraclavicular areas were free of palpable lymphadenopathy. Examination of her heart revealed no murmur and signs of failure. The lungs were bilaterally clear to auscultation. The breasts exhibited no apparent asymmetry and deformity. A thorough musculoskeletal examination shown full range of motion in joints, no tenderness over spine, and negative FABER/FADIR tests. There was no evidence of skin changes, focal motor weakness, sensory deficits, or arthritis.

## 3 METHODS

**FIGURE 1:**
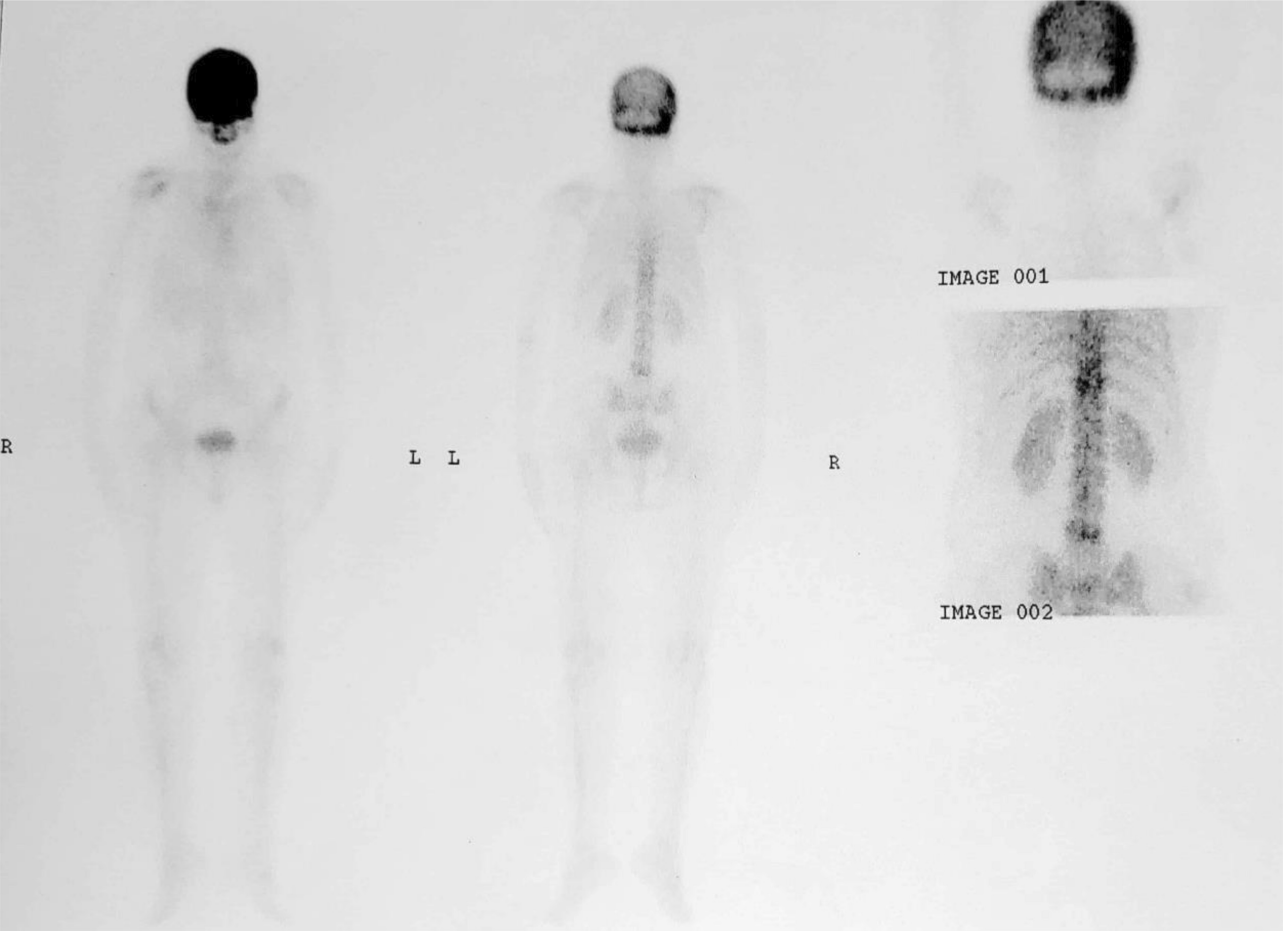
Bone scintigraphy by Tc‐99m‐MDP showing increased tracer activity in the skull, T11 and L5 vertebrae which is highly suggestive of Paget's disease of bone. Although Tc‐99m‐MDP is highly sensitive but not specific. It is useful to define the overall extent and distribution of disease.

**FIGURE 2:**
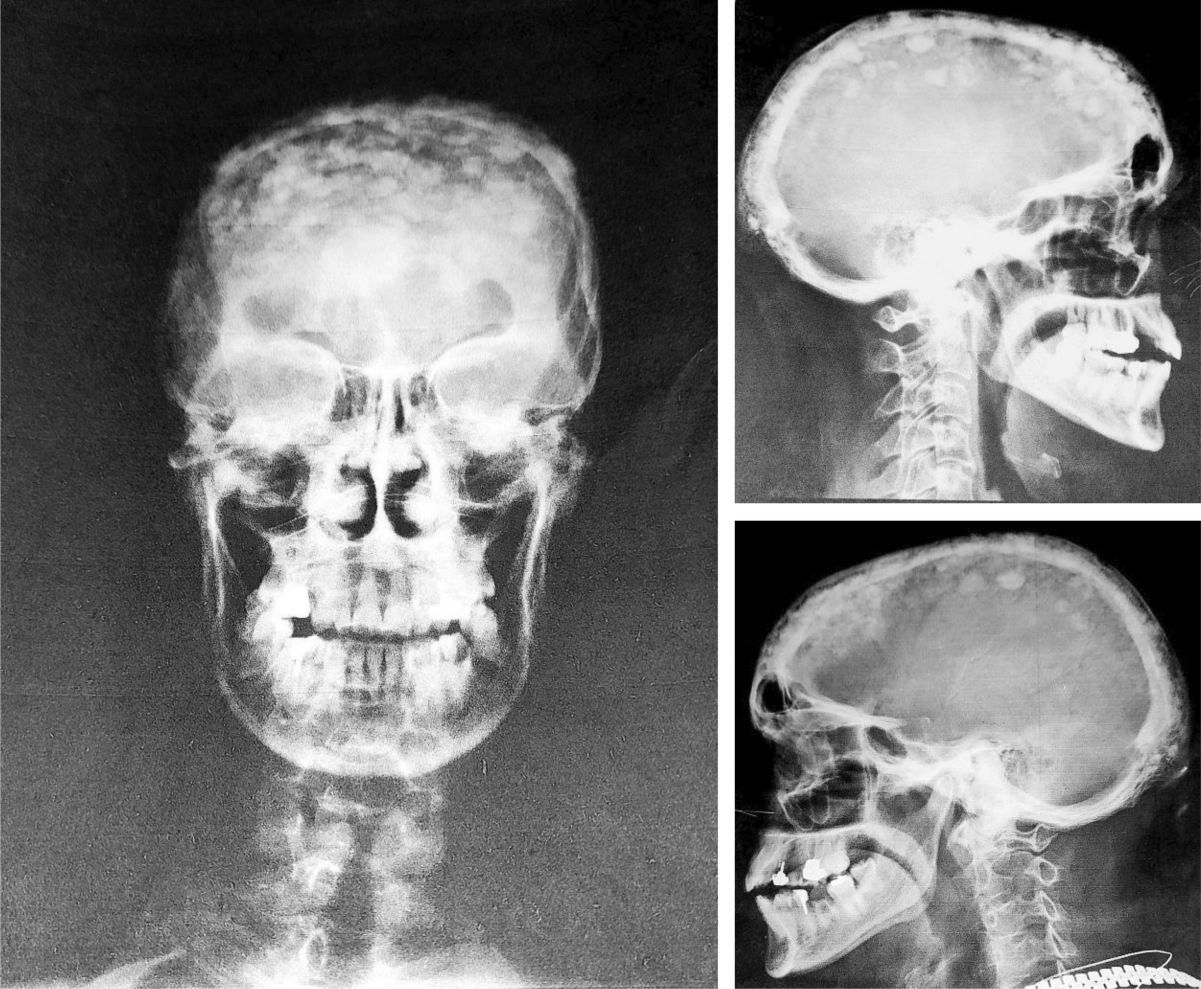
Plain radiography of skull showing poorly defined and fluffy sclerotic patches along with lytic lesions known as cotton wool appearance a classic sign of Paget's disease of bone.

In the prior laboratory workups, the complete blood count revealed red‐cell count 5.07 × 10^6^ per microlitre (reference range 3.9–5.8); hemoglobin 15.1 g per decilitre (reference range 12–16); mean corpuscular volume 85 femtolitre (reference range 80–100); mean corpuscular hemoglobin 29 pg (reference range 27–32); white‐cell count 7300 per microlitre (reference range 3500–10,000) with differential count of 56% neutrophils, 37% lymphocytes, 4% monocytes, and 3% eosinophils; platelet count 283,000 per microlitre (reference range 150,000–450,000); the serum level of ALP was 2414 U per liter (reference range, 64–306) along with gamma‐glutamyl transferase (GGT) of 17 U per liter (reference value, ≤32); total bilirubin of 0.8 mg per decilitre (reference range 0.1–1.2) and direct portion of 0.4 mg per decilitre (reference range 0.1–0.3). During laboratory reassessment, serum ALP level was 2933 U per liter (bone‐specific isoform of ALP was not available); intact parathyroid hormone (iPTH) 18.2 pg per milliliter (reference range, 10–65); calcium 9.4 mg per decilitre (reference range, 8.5–11); phosphorus 3.6 mg per decilitre (reference range, 2.5–5); other laboratory test results are shown in Tables 1 and 2. Evaluation followed by a breast ultrasonography which revealed fibrocystic changes and bilateral reactive lymph nodes (BI‐RADS 0). Bone mineral density (BMD) using dual energy x‐ray absorptiometry (DXA) shown worst T‐score of −2.9 in AP spine and −2.7 in femoral neck. The patient's 10‐year risk of fracture as calculated by FRAX for major osteoporotic fracture as 8.5% and for hip fracture as 1.6%. A whole bone scan following injection of 20 mCi Tc‐99 m‐MDP in the anterior and posterior projections showed diffusely increased isotope uptake in the skull, T11, and L5 vertebrae (Figure 1). Plain radiography showed mixed lytic and sclerotic lesions of the skull, consistent with cotton wool appearance (Figure 2). The diagnosis of Paget's disease of bone has been placed.

**TABLE 1:** Basic metabolic panel and inflammatory markers.

Variable	Reference range	2 months before current evaluation	1 week before current evaluation
Fasting glucose	70–100	122	—
Urea (mg/dL)	21–45	34	—
Creatinine (mg/dL)	0.6–1.4	1.2	—
Erythrocyte sedimentation rate (mm/h)	0–20	—	13
C‐reactive protein (mg/L)	< 10	—	2

**TABLE 2:** Liver function tests.

Variable	Reference Range	2 months before current evaluation	2 weeks before current evaluation	1 week before current evaluation	On current evaluation
Aspartate aminotransferase (U/L)	0–31	21	22	26	—
Alanine aminotransferase (U/L)	0–31	17	16	16	—
Alkaline phosphatase (U/L)	64–306	2400	3060	2414	2933

Although the patient was asymptomatic, treatment initiated by Alendronate 70 mg two doses per week to suppress the activity of the disease and to prevent its further progress.

**FIGURE 3:**
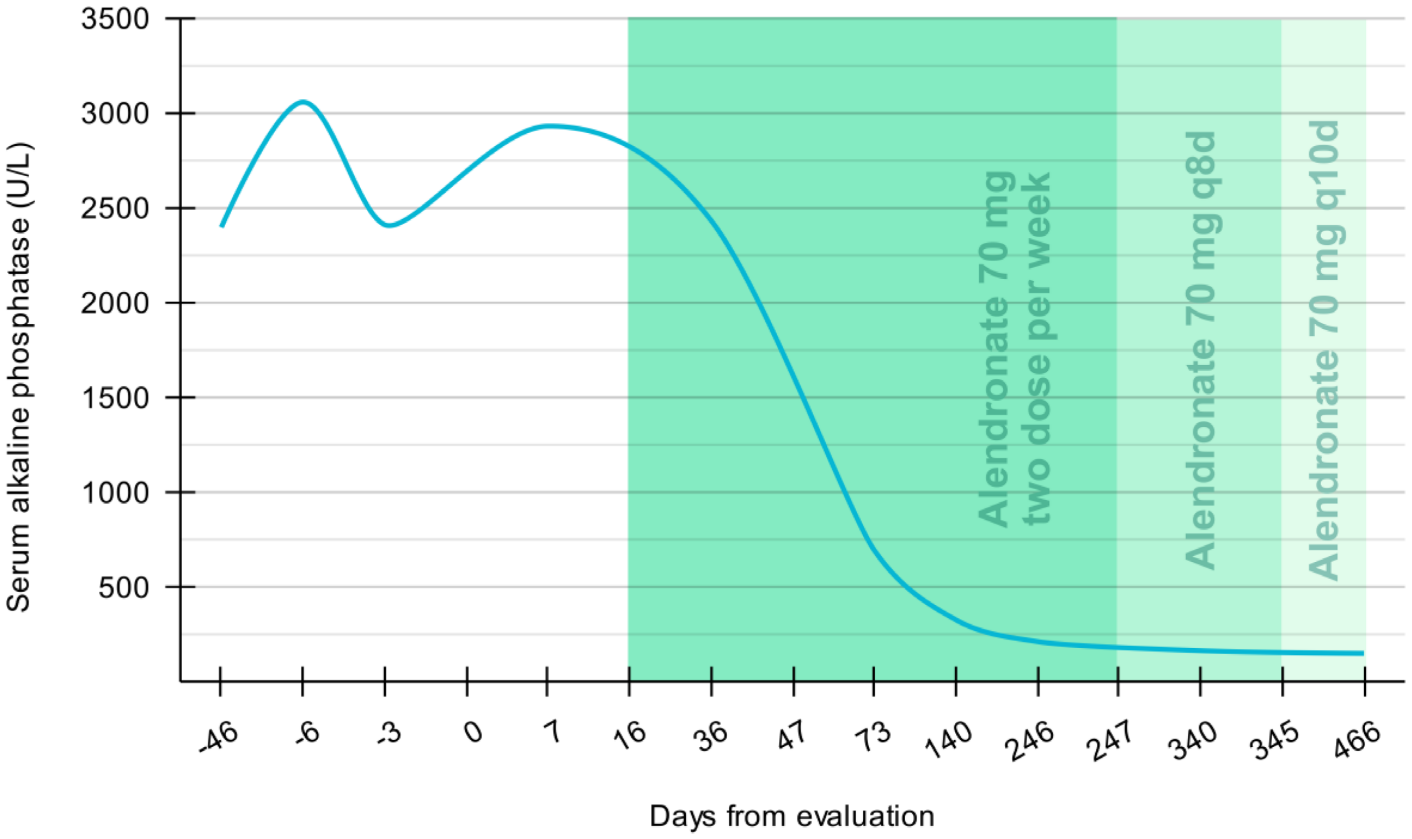
Trend of serum ALP levels as a biochemical marker of disease activity during the course of management.

**FIGURE 4:**
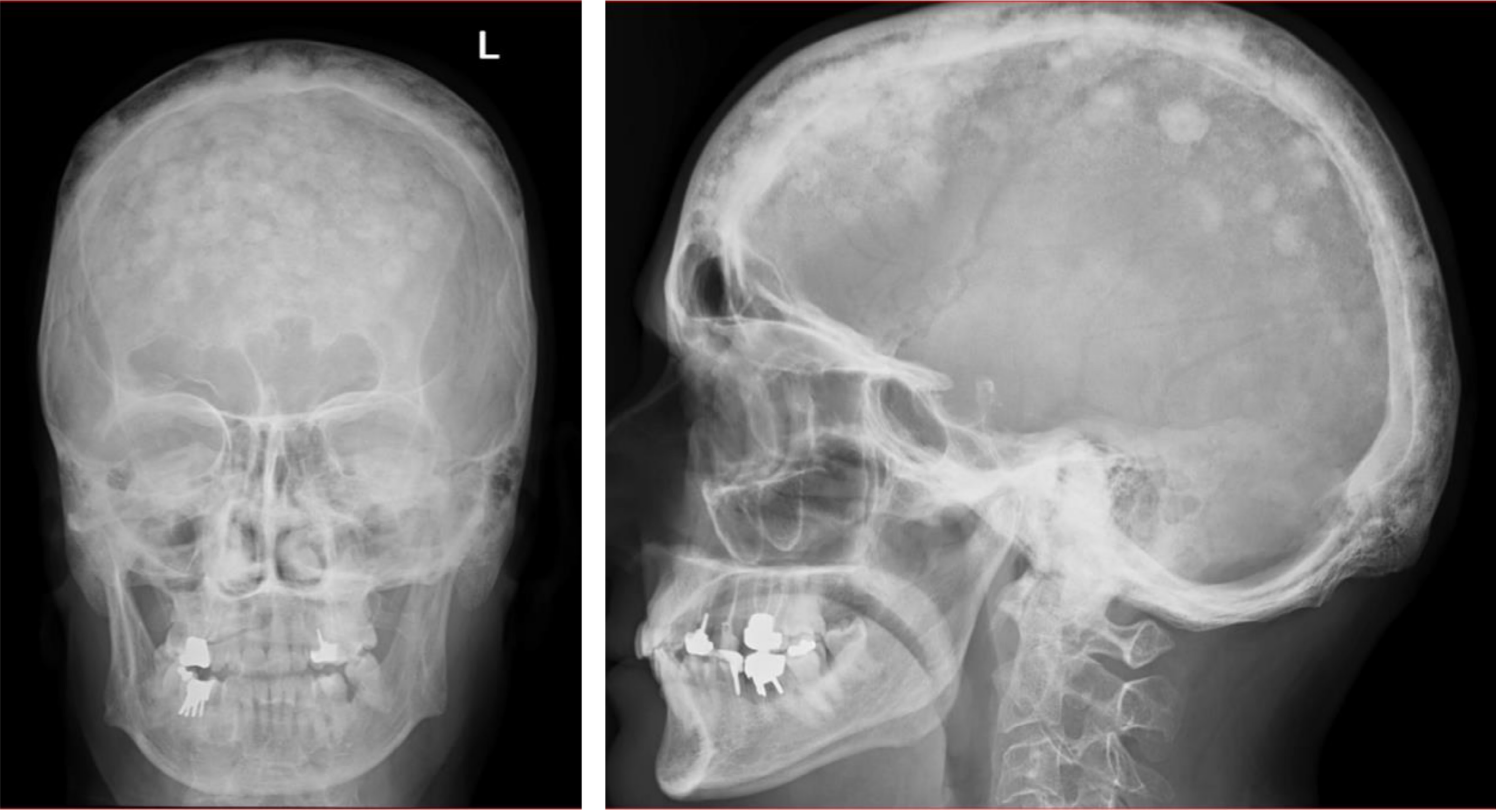
Plain radiography of skull 20 month after treatment showing marked improvement of lytic and fibrotic lesions.

During the follow‐up visit the next month, the patient reported mild pain in her knees, ankles, and tingling in her feet, which spontaneously dissipated in subsequent visits. Disease activity, judged biochemically by serum ALP levels during follow‐ups (Figure 3). During 8 months, the serum ALP level dropped to 216 U per liter. Subsequently, the dose of Alendronate was reduced to 70 mg every 8 days for 4 months, and then followed by 70 mg every 10 days as the BMD was strongly suggestive of osteoporosis in the context of Paget's disease of bone. A control plain radiography of skull 20 month following treatment shown marked improvement of lesions (Figure 4).

## 4 CONCLUSION AND RESULTS

This presentation elucidates the diagnostic and therapeutic approaches employed in addressing Paget's disease of the bone in a 57‐year‐old female patient exhibiting elevated alkaline phosphatase levels in a nonendemic locale. Despite the absence of overt symptoms, a regimen involving an unconventional dosage of Alendronate was initiated to mitigate disease activity and impede its progression. Encouragingly, the patient exhibited a favorable response to the treatment, as evidenced by a gradual decline in serum ALP levels over the course of treatment. Regular follow‐up evaluations and meticulous monitoring of biochemical markers play a pivotal role in evaluating disease activity and tailoring treatment strategies accordingly. This case underscores the paramount importance of early detection and timely intervention in effectively managing Paget's disease.

## 5 DISCUSSION

In the management of Paget's disease, the selection of treatment modalities is contingent upon the specific clinical manifestations exhibited by the patient. Therapeutic options encompass a spectrum of interventions ranging from pharmaceutical agents to surgical interventions. A pivotal aspect involves the timely initiation of therapeutic interventions aimed at alleviating localized bone pain within the affected anatomical site. Pharmacological strategies encompass a repertoire of medications including analgesics, bisphosphonates, and calcitonin, among others.^13^ Notably, bisphosphonates demonstrate consistent efficacy in mitigating markers of bone resorption in active Paget's disease, consequently eliciting a subsequent reduction in markers indicative of bone formation, notably alkaline phosphatase (ALP), which serves as a widely recognized indicator of metabolic activity. Detailed guidelines pertaining to the administration and dosage regimens of this pharmacotherapeutic class are delineated in Table 3.^2^, ^14^

**TABLE 3:** Dosages of bisphosphonate for the treatment of PDB.^15^

Drug	Route of administration and dose	Posology
Zoledronic acid	5 mg‐intravenous	Single infusion
Pamidronate	60 mg‐intravenous	Three infusions
Risedronate	30 mg/day‐oral	2 months
Alendronate	40 mg/day‐oral	2–6 months
Tiludronate	400 mg/day‐oral	3–6 months
Etidronate	400 mg/day‐oral	3–6 months

Alendronate (alendronic acid; 4‐amino‐1‐hydroxybutylidene bisphosphonate) has shown oral efficacy in managing Paget's disease of the bone. Through its interaction with hydroxyapatite crystals in bone, alendronate inhibits osteoclast‐mediated bone resorption, thereby reducing breakdown of the bone matrix. These dual mechanisms play a pivotal role in regulating mineral reabsorption and turnover. The oral bioavailability of alendronate ranges from 0.9% to 1.8%, with food significantly impacting its absorption. The elimination of the drug from bone mirrors the underlying skeletal turnover rate. Renal clearance is believed to involve both glomerular filtration and a specialized secretory pathway.^15^, ^16^

The variation in our treatment program can be attributed to several factors associated with the countries within the region, gender, and etiology of Paget's disease. Epidemiological data indicates a higher prevalence of this disease in Northern Europe.^4^ However, we have encountered a case of Paget's disease in an Asian country, which is considered a nonendemic region for this particular ailment. This discrepancy justifies the implementation of a distinct treatment plan tailored to the unique circumstances of this case.

Furthermore, Paget's disease is predominantly observed in individuals aged 55 and above, with a higher incidence among men.^4^ However, the case under consideration involves a woman, highlighting another noteworthy distinction. Additionally, the etiology of Paget's disease encompasses a broad and still unknown spectrum, encompassing potential genetic and viral causes.^4^, ^5^ The potential implication of the Measles virus (MV) or other paramyxoviruses as etiological factors in Paget's disease is a subject of ongoing scientific discourse. Notably, several studies have reported the detection of paramyxovirus antigens within the osteoclasts of afflicted individuals. The case of our examined patient, characterized by a history of measles, lends additional credence to the hypothesis of viral involvement in the pathogenesis of Paget's disease.^17^, ^18^ Given the absence of a definitive diagnosis regarding the underlying cause of this disease in our specific case, it can be argued that the differing etiology is an additional contributing factor to the variance in treatment approaches.

Different local conflicts can cause symptoms such as bone pain, headache, hearing loss, etc. In order to reduce these symptoms, treatments are inevitably considered.^8^, ^9^ After considering the various options, it is evident that our treatment and drug dosage differ in this case, as there can be different conflicts in each person.

Bisphosphonates exhibit varying levels of bioavailability depending on the route of administration (oral or injection) and the frequency of consumption per week. When taken orally, continuous usage with a low dosage may lead to gastrointestinal complications in the esophagus. Conversely, a higher dosage administered over fewer days can mitigate these complications. The drug is absorbed by bones with a high turnover rate and remains within the bone until the bone deposit is eliminated, a process that can span from 1 to 10 years.^19^, ^20^ Consequently, the dosage and continued usage of this drug in the present scenario can be rationalized to a certain extent.

A diverse range of oral alendronate treatment regimens, as opposed to paget zoledronic acid, have been extensively studied in the treatment of paget disease and have demonstrated comparable efficacy. Regarding the dosage of alendronate utilized in our case, numerous published studies have employed doses ranging from 40 to 50 mg per day, with varying levels of adherence and generally satisfactory clinical and biological outcomes. Following the patient's disclosure of occasional transient and occasionally incapacitating side effects associated with intravenous zoledronic acid, the patient expressed a preference for oral bisphosphonate therapy. In the context of utilizing Fosamax (70 mg alendronate tablets), our recommendation was to administer two tablets twice weekly for the initial 3 months to enhance patient compliance and address this matter comprehensively. This approach was considered in the context of managing mild to moderate Paget's disease of the bone.^21^, ^22^

## AUTHOR CONTRIBUTIONS

**Nasrin Razavianzadeh:** Project administration; supervision. **Soheil Shahramirad:** Investigation; writing – original draft. **Mohammad Hasani:** Formal analysis; writing – original draft. **Hessamedin Babaei:** Investigation; methodology; writing – original draft.

## FUNDING INFORMATION

None.

## CONFLICT OF INTEREST STATEMENT

The authors declare that they have no financial or personal conflicts of interest associated with this case report.

## ETHICS STATEMENT

The authors declare that this submission follows the policies of Clinical Case Reports as outlined in the Guide for authors and in the Ethical Statement.

## CONSENT

Written informed consent was obtained from the patient to publish this report in accordance with the journal's patient consent policy.

## Data Availability

The data that support the findings of this study are available on request from the corresponding author. The data are not publicly available due to privacy or ethical restrictions.
